# The Relation Between Classroom Setting and ADHD Behavior in Children With ADHD Compared to Typically Developing Peers

**DOI:** 10.1177/10870547231167522

**Published:** 2023-04-11

**Authors:** Anouck I. Staff, Jaap Oosterlaan, Saskia van der Oord, Barbara J. van den Hoofdakker, Marjolein Luman

**Affiliations:** 1 Vrije Universiteit Amsterdam, The Netherlands; 2 University of Amsterdam, The Netherlands; 3 KU Leuven, Belgium; 4 University of Groningen, The Netherlands; 5 Accare Child Study Centre, Groningen, The Netherlands

**Keywords:** ADHD, classroom observations, classroom setting, transitions

## Abstract

**Objective:**

More knowledge on the impact of classroom setting on behavior of children with ADHD may help us to better adjust classroom settings to the needs of this group.

**Method:**

We observed ADHD behaviors of 55 children with ADHD and 34 typically developing peers (6–12 years) during classroom transitions, group lessons, and individual seatwork.

**Results:**

Multivariate analyses revealed that levels of motor and verbal hyperactivity increased during classroom transitions compared to group lessons and individual seatwork. Children in the ADHD group were more off-task, across settings. There were no interactions between group and setting.

**Conclusions:**

Children with ADHD were similarly affected by classroom setting compared to typically developing peers, despite being more off-task across settings. Further research into whether the observed increase in hyperactivity during classroom transitions may be problematic or possibly even beneficial for children with ADHD is recommended.

## Introduction

Inattentive, hyperactive, and impulsive behaviors of children with ADHD often exacerbate at school because of the special demands placed on self-regulation and motivation. These behaviors may be reason for clinical referral (Pelham et al., 2005) and may lead to impairments in academic (e.g., grades) as well as non-academic (e.g., peer problems) school functioning for children with both subthreshold and full threshold ADHD (Zendarski et al., 2022). Furthermore, teachers experience difficulties managing these behaviors (Greene et al., 2002). Given that approximately 5% of all school-aged children fulfill diagnostic criteria for ADHD (Polanczyk et al., 2014) and another 11% to 18% of the children experience impairing symptoms without meeting full diagnostic criteria (i.e., subthreshold ADHD) (Kirova et al., 2019), it is important to gain insight into classroom settings that may ameliorate or worsen the problems that these children experience in the classroom. This knowledge may contribute to adjusting classroom settings to the needs of children with ADHD and support teachers in how to deal with ADHD behaviors in the classroom.

Frequently occurring classroom settings include individual seatwork and group lessons, alternated with classroom transitions. These settings place different demands on children, and depending on characteristics of the child (e.g., self-regulation and motivation) and teacher (e.g., amount of structure, direction, and feedback), as well as environmental factors that provide learning opportunities (e.g., modeling, vicarious learning); these demands may differentially affect classroom functioning. In group lessons (e.g., class wide instructions, each child works on the same task at the same time), teachers usually structure the environment which may be beneficial for children because less self-regulation is required. Group lessons may also provide children with possibilities to learn from other children what behavior is expected, for example by seeing or hearing what other children do (modeling) (Bandura, 1980; Schunk, 1987), and/or by seeing or hearing what behavior of other students is rewarded by the teacher (vicarious learning) (Bandura, 1965; Kazdin, 1979). However, group lessons also require passive engagement (e.g., listening to teacher instructions) that may decrease motivation and may lead to children being easily distracted (Finn et al., 2003; Junod et al., 2006). During individual seatwork, children are required to work independently while teacher structure, direction, and feedback is lower compared to group lessons. The required level of the child’s self-regulatory skills as well as intrinsic motivation may therefore be higher (Stright & Supplee, 2002) and contribute to off-task and hyperactive behavior (Linnenbrink & Pintrich, 2002). Finally, during classroom transitions, children have to stop their current activity and perform a long chain of tasks, with often periods of waiting in between. During these transitions, teachers usually provide instructions in multiple steps at once, placing large demands on children’s self-regulation (e.g., switching between tasks). At the same time, children may face delays that may decrease motivation (Carbone, 2001; McIntosh et al., 2004; Reid, 1999). These demands placed on self-regulation and motivation are expected to result in higher levels of off-task and hyperactive behavior during classroom transitions (Antrop et al., 2005).

For children with ADHD, it may be particularly difficult to adapt their behavior to the demands of the different classroom settings. These children often experience difficulties in several neurocognitive domains that are important for self-regulation and motivation (like inhibition, working memory, intrinsic motivation, and reinforcement learning; Faraone et al., 2015), and they show difficulties with waiting (Sonuga-Barke et al., 2008; Sonuga-Barke, Wiersema, et al., 2010). We hypothesized that behavior of children with ADHD may exacerbate in situations that place a high demand on self-regulatory processes, such as unstructured situations (e.g., transition moments; Zentall & Leib, 1985), as well as situations that may be experienced as not motivating (e.g., low stimulation or delays during group lessons; Antrop et al., 2000, 2006; Rimm-Kaufman et al., 2005).

Although there is a rich history of structured behavioral observations of children with ADHD in classroom settings (e.g., Abikoff et al., 1977, 1980; Atkins et al., 1985; Kofler et al., 2008; Volpe et al., 2005), so far only few studies have looked into the relationship between classroom settings and ADHD behaviors (i.e., inattention, hyperactivity). Furthermore, although some studies compared multiple classroom settings among which classroom transitions (e.g., Coelho et al., 2019; Rimm-Kaufman et al., 2005; Vitiello et al., 2012), those classroom transitions have not yet been taken into account in studies comparing children with ADHD to typically developing children, nor have there been any head-to-head comparisons between all three settings (i.e., individual seatwork, group lessons, and classroom transitions). In general, there are some indications that group lessons are associated with lower student engagement (Coelho et al., 2019; Rimm-Kaufman et al., 2005), but these studies have not included children with ADHD. The evidence of studies that compared children with ADHD to typically developing children on inattentive behavior in different settings is mixed. Two studies found that children with ADHD showed higher levels of off-task behavior and lower levels of engagement during group lessons compared to other settings, including individual seatwork (Steiner et al., 2014; Zentall, 1980). For “other settings,” that consisted predominantly of individual seatwork, but also included working in small groups, there were no group differences on inattentive behavior. However, this finding may have been affected by taking individual seatwork and small group work together in one coding category, because small groups are shown to be beneficial for on-task behavior in children with ADHD (Baker et al., 2008; Hart et al., 2011). Two other studies (Imeraj et al., 2013; Lauth et al., 2006) did not report on group by setting interactions for inattentive behavior. Imeraj et al. (2013) observed that, when group lessons were compared to individual seatwork, children with ADHD showed more inattentive behavior than typically developing peers despite setting. Also Lauth et al. (2006) reported more inattentive behavior in children with ADHD, but they found that inattentive behavior of all children (despite group status) was highest during group lessons compared to other settings. Further, also regarding hyperactive behaviors of children with ADHD and typically developing children in different settings, results are mixed. All studies observed higher levels of hyperactive behavior in children with ADHD (Lauth et al., 2006; Steiner et al., 2014; Zentall, 1980). But whereas hyperactivity levels were similar across settings in the study by Steiner et al. (2014), the other two studies showed higher levels of hyperactivity during individual seatwork compared to group lessons. Nevertheless, none of the studies found group by setting interactions. Taken together, the few available studies that observed behavior during group lessons and individual seatwork in children with ADHD showed heterogenous findings (Imeraj et al., 2013; Lauth et al., 2006; Steiner et al., 2014; Zentall, 1980), therefore, more studies comparing inattentive and hyperactive behavior of children with ADHD and typically developing children during different classroom settings are needed.

For classroom transitions, there are indications that setting has a differential effect on children with ADHD and typically developing children. These moments occur multiple times during a school day (i.e., could take up to 30% of the day, Codding & Smyth 2008; Schmit et al. 2000) and transitions seem inversely related to student’s inattentive and disruptive behavior (Cameron et al., 2005; Codding & Smyth, 2008). For children with ADHD, classroom transitions may further increase motor and verbal hyperactivity as compared to typically developing children, as these moments often include idle time or waiting (Antrop et al., 2005). A study on idle time (i.e., moments where children have no task to perform, which has some overlap with transition moments) revealed that motor and verbal hyperactivity of all children increased during idle time compared to non-idle time, but that this increase was larger in children with ADHD compared to typically developing classmates (Imeraj et al., 2016). By observing ADHD behavior of children with ADHD as compared to typically developing children during all three classroom settings, the current study can provide insight into how these settings relate to each other. Furthermore, by examining both inattentive and (verbal and motor) hyperactive behavior we will be able to see if these behaviors may compensate for each other (e.g., hyperactivity may have a functional role to counteract inattention; Sarver et al. (2015)).

Thus, the current study aimed to gain insight into the relation between classroom setting and ADHD behaviors of children in order to get more insight into how to optimally adapt classroom setting to their needs. Off-task behavior, motor hyperactivity and verbal hyperactivity of children with ADHD and typically developing children were compared during three common classroom settings (i.e., group lessons, individual seatwork, classroom transitions). We examined whether off-task behavior and hyperactivity of children increased during classroom transitions as compared to group lessons or individual seatwork, and whether this increase was larger in children with ADHD as compared to typically developing children (Antrop et al., 2005; Imeraj et al., 2013, 2016). There are some indications that group lessons have a detrimental effect on on-task behavior (Rimm-Kaufman et al., 2005).

## Method

### Participants

Participants were 55 children with (subthreshold) ADHD (referred to as ADHD group) and 34 typically developing children (referred to as controls). Children were 6 to 12 years old and attended regular primary education (grade 1–6).

Children from the ADHD group were recruited as part of an intervention study on behavioral teacher training (see Staff et al., 2021). Inclusion criteria were: (a) high levels of ADHD symptoms (>90th percentile) as rated by teachers on the Inattention and/or Hyperactivity/Impulsivity scale of the Disruptive Behavior Disorders Rating Scale (DBDRS) (Oosterlaan et al., 2008; Pelham et al., 1992), (b) at least three symptoms (item score ≥2) on the Inattention and/or Hyperactivity/Impulsivity scale of the semi-structured Teacher Telephone Interview (TTI) (Tannock et al., 2002), based on the DSM-IV-TR (American Psychiatric Association, 2000), and (c) a score >5 (indicating classroom-related impairment, range 0–10) on at least one domain of functioning on an adapted version of the teacher rated Impairment Rating Scale (IRS) (Fabiano et al., 2006). In addition, children included in the ADHD group were required to obtain a score >80th percentile on the Hyperactivity scale of the teacher-rated Strengths and Difficulties Questionnaire (SDQ) in order to avoid overlap between the ADHD and control group (see below) in terms of ADHD symptoms.

Children in the control group were classmates of children in the ADHD group (85% of children) or were recruited otherwise (e.g., through advertisements in school newsletters). Inclusion criteria were: (a) low levels of ADHD symptoms as assessed with a score <80^th^ percentile on the Hyperactivity scale of the teacher-rated SDQ (Diepenmaat et al., 2014; Goodman, 1997), and (b) not attending special education classes.

In addition, children in both groups were excluded if they: (a) had an estimated full scale IQ < 70, estimated using a short version of the Wechsler Intelligence Scale for Children-third edition (WISC-III-NL) (Wechsler et al., 2005), (b) were currently taking psychotropic medication or during the last month, or (c) had been clinically diagnosed with autism spectrum disorder or conduct disorder according to the DSM-IV-TR (American Psychiatric Association [APA], 2000) or DSM-5 (APA, 2013) as reported by parents.

### Materials

#### Observational Measure

In order to measure ADHD behaviors as well as classroom setting, observations were conducted when children attended morning lessons in their own classroom led by their primary teacher (with the exception of four children, two in each group, of whom observations were collected in the afternoon due to practical reasons). Children were observed for approximately 90 minutes. When children in the ADHD and control group were classmates, they were observed simultaneously using two video cameras. Videos of the classroom observations were coded and coders were allowed to stop and rewatch the video. We selected the first 60 minutes from these 90 minutes that contained actual lessons (e.g., the observation started when children were arriving at the beginning of the day, coding started when the teacher started the first lesson). Coding time differed somewhat between observations, because for some observations less than 60 minutes of actual lessons were available, while others contained a little over 60 minutes of lessons (total time coded: *M* = 1:02 hr, *SD* = 0:06 hr).

Observations were coded using the behavioral Attention Problems (i.e., visual attention to task), Motor Hyperactivity (i.e., motor movements) and Verbal Hyperactivity (i.e., talking, other vocalizations) scales, as well as the Classroom Setting scale of Ghent University Classroom Coding Inventory (GUCCI; Imeraj et al., 2013, 2016; Staff, Oosterlaan, Imeraj, et al., 2020). We also coded Oppositional Behavior, but this scale was excluded for the current study given the low rates of observed oppositional behavior (*M* = 0.87, *SD* = 2.11 for the ADHD group; *M* = 0.00, *SD* < 0.001 for the control group). Each behavioral scale comprises a mutually exclusive, categorical variable of behavior to be coded as absent or present (e.g., Verbal Hyperactivity scale distinguished between the categories no verbal hyperactivity and verbal hyperactivity), see Table 1. Behaviors were continuously coded; that is, behaviors were coded throughout the coding period. Outcome was the proportion of time behavior occurred, calculated per setting. Psychometric properties of the GUCCI have been shown to be adequate in a previous study (see for a detailed description: Staff, Oosterlaan, Imeraj, et al., 2020). In short, associations between scales are small to moderately-sized (*r* = −.07 to .44). Inter-observer agreement (ICC) was excellent for all behavioral scales (*ICC* > 0.78) and all scales have been shown successful in discriminating between children with ADHD and typically developing children across settings (but not differentiated between settings). Convergent validity as compared to teacher ratings and clinical interview was adequate for the Verbal Hyperactivity scale (*ρ* = .26 to .28), but less clear (*ρ* = .01 to .19) for Attention Problems and Motor Hyperactivity, although this is in line with meta-analytic findings (Staff, Oosterlaan, van der Oord, et al., 2020).

**Table 1.** Operational Definitions of Observed Behaviors and Settings.

Scale	Coding category	Description	Metric
Child behaviors			
Attention problems	On-task	The child is involved in activities that are expected by the teacher (e.g., paying visual attention to task or to the teacher), and is following teacher’s instructions and requests.	
	Off-task	The child is involved in activities that are not expected by the teacher for at least 2 s (e.g., not working on assignments, daydreaming). During transitions this included children not following teacher’s instructions and requests.	% of time
Motor hyperactivity	No motor hyperactivity	The child remains seated. Small, but not disturbing, movements of arms, hands, feet, or legs are observed.	
	Motor hyperactivity	The child is not sitting (still) on the chair (e.g., overturns or swings the chair, standing up without permission, walks through the classroom). The child shows small or large movements that are annoying or disturbing peers (e.g., tapping with a pen, pricking neighbor with a finger).	% of time
Verbal hyperactivity	No verbal hyperactivity	The child is quiet, or the child talks in reaction to the teacher’s request.	
	Verbal hyperactivity	The child is talking or making vocal sounds (e.g., whispering to self, humming).	% of time
Context variables			
Classroom setting	Group lessons	The teacher gives academic instructions or provides class wide teacher feedback, or each child is supposed to be working at the same time on the same task.	% of time
	Individual seatwork	Students work individually without ongoing teacher instructions.	% of time
	Classroom transitions	Teacher provides instructions on tidying up stuff or preparing for lessons, which may be for the whole group or for the target student. These are often periods between two different lessons or periods just before or after a break.	% of time

We coded three possible classroom settings (see Table 1): *group lessons, individual seatwork*, and *classroom transitions*. “Other” was coded during all other settings, such as small group instructions with less than six students or when the child was excused from the classroom, for example to go to the toilet. The “other” category was excluded from the current analyses. The Classroom Setting scale was not mutually exclusive. Group lessons and individual seatwork were coded for the whole class; length of transition moments was individually coded, because these may differ per child. Classroom transitions included moments of instructions for the whole group (e.g., usually the introduction of the transition moment) as well as moments of individual seatwork in which the observed child was preparing to work independently (e.g., transition time for this particular child ended when he/she started working on the individual task).

Behavioral scales and classroom setting were coded using Observer XT Software (Zimmerman et al., 2009), allowing us to import video material, code behaviors, and calculate outcomes. The percentage of the time each of the child behaviors occurred were calculated separately for group lessons, individual seatwork and classroom transitions. Observations were coded by independent observers (i.e., trained graduate students) who were unaware of group status of the child. Observers were individually trained by the first author (AS) and observations were coded according to a manual providing in depth instructions on how to code behaviors supplemented with detailed examples. For more information on the coding procedures, training of observers and psychometric properties of the GUCCI, please see Staff, Oosterlaan, Imeraj, et al. (2020).

#### Rating scale

ADHD symptoms were assessed using the Hyperactivity scale of the SDQ, completed by teachers. This scale consists of five items measuring inattention and hyperactivity/impulsivity on a 3-point Likert scale (0 = *not true*, 1 = *somewhat true*, 2 = *certainly true*). Scores may range from 0 to 10, with higher scores indicating more ADHD symptoms. The internal consistency of this scale is high (α = .89; Van Widenfelt et al., 2003) and convergent validity is strong (Stone et al., 2015).

### Procedure

Teachers were recruited through school principals, educational consult associations, and an outpatient mental health clinic. Participating teachers enlisted one to two children displaying ADHD symptoms for which they needed behavioral support. Typically developing children were recruited by asking participating teachers to enlist a typically developing control child of the same age. Consent was obtained from teachers, parents and children older than 11 years. Data collection (i.e., classroom observations, WISC-III and teacher- and parent questionnaires) occurred during one week. Teachers introduced observers as interns who visited different classes for their study, to observe how children are working during lessons. Cameras were positioned in a corner in front of the classroom to prevent target children being aware that they were the object of observation. Parents and teachers agreed with this procedure. The study was conducted in accordance with the Declaration of Helsinki (World Medical Association, 2013) and the local medical ethical committee waived the need for medical ethical approval (University Medical Centre Groningen, 2016/198).

### Statistical analysis

Stata (version 16) was used for data analysis. Groups were compared on demographic characteristics using independent samples *t*-tests and chi-square tests, and if differences were observed, analyses were repeated with groups matched on these variables. Outliers (>3SD) were winsorized (Tabachnick et al., 2007). Missing data was <5% for all outcomes, missing data was not imputed. Because children in both groups were classmates, multivariate mixed model analyses were used to compare groups on behavioral outcomes during the different settings. Four hierarchical levels were distinguished: observations (level 1), nested within children (level 2), nested in classrooms (level 3), and nested in schools (level 4). Random intercepts at classroom and school level were included only if they significantly improved model fit as determined by Likelihood Ratio Tests. We inserted group as between-subject factor (*ADHD* = 0, *control* = 1) and setting as within-subject factor (*group lessons* = 0, *individual seatwork* = 1 and *classroom transitions* = 2) to look at group by setting interactions. We also conducted sensitivity analyses comparing only children in the ADHD group without parent-reported clinical ADHD diagnosis to the control group, in order to examine whether effects held up for the subthreshold ADHD group. Effect sizes were expressed as Cohen’s *d*, with .20, .50, and .80 referring to the thresholds for small, medium and large effects, respectively, or Cramer’s *V* with .07 to .21 referring to small, .21 to .35 to medium, and >.35 to large effects (Cohen, 2013).

### Power Analysis

We conducted the power analysis using G*Power. To test for group differences and group by setting interactions, we used a multivariate repeated measures ANOVA with a between group-factor (children with ADHD and controls) and a within-group factor (three settings). Based on the mixed results of the studies performed so far (Imeraj et al., 2013; Lauth et al., 2006; Steiner et al., 2014; Zentall, 1980) we expected a small to medium sized effect (*f* = .20) for the group by setting interaction, and a small to medium correlation between settings (*r* = .30). Power analysis revealed that a total number of 58 participants (α = .05, power = .80) would be sufficient to test this effect. We aimed to include 10% more children to account for nesting (four levels) in the mixed models (Twisk, 2021), thus a total of 64 children.

## Results

Sample characteristics are presented in Table 2. Results revealed no differences in age and IQ between the ADHD and control group, but, as expected, higher rates of teacher and parent rated ADHD in the ADHD group compared to controls (*d* = 1.03–4.53, see Table 2). As expected based on the higher rates of ADHD symptomatology in boys (Willcutt, 2012), there were more boys in the ADHD group compared to the control group. All analyses were re-run using groups matched on sex (see Supplemental Table S1).

**Table 2.** Sample Characteristics.

	ADHD (*n* = 55)	Control (*n* = 34)	Group comparisons
Age (years)	8.55 (1.32)	8.76 (1.54)	*t*(87) = *−*.67, *p* = .502, *d* = .15
Sex, *n* (%) boys	44 (80.0)	17 (50.0)	χ^2^(1) = 8.77, *p* = .003, *V* = .31
IQ	100.02 (11.83)	105.20 (15.21)	*t*(83) = *−*.17, *p* = .085, *d* = .39
Race, *n* (%) Caucasian	3 (5.45)	4 (11.76)	*χ*^2^(1) = 1.16, *p* = .283, *V* = .11
TTI (number of symptoms)			
Inattention	4.62 (1.68)		
Hyperactivity/impulsivity	4.02 (2.13)		
ODD	.91 (1.27)		
IRS (scale 0–10)			
Number of domains	3.16 (.97)		
SDQ (scale 0–10)			
Hyperactivity	8.51 (1.33)	.59 (.82)	*t*(87) = *−*.34.72, *p* < .001, *d* = .6.81
Setting duration (% of total observation)			
Group lessons	52.66 (19.01)	47.91 (20.93)	*t*(83) = 1.08, *p* = .283, *d* = .24
Individual seatwork	42.81 (22.83)	44.18 (25.52)	*t*(82) = *−*.26, *p* = .799, *d* = .07
Classroom transitions	11.12 (6.86)	12.45 (7.58)	*t*(79) = *−*.81, *p* = .421, *d* = .19

*Note. M* and *SD* are depicted unless otherwise stated.

ADHD = attention-deficit/hyperactivity disorder; IRS = Impairment Rating Scale; ODD=oppositional defiant disorder; SDQ = Strengths and Difficulties Questionnaire; TTI = Teacher Telephone Interview.

In the ADHD group, 14 of the 55 children (25%) had been clinically diagnosed with ADHD and 26 children (47%) met the threshold for a diagnosis of ADHD based on the TTI. None of children in the ADHD group had received a diagnosis of ODD, but two children (4%) met ODD criteria as indicated on the TTI.

### Observed ADHD Behavior Across Settings

**Figure 1.**
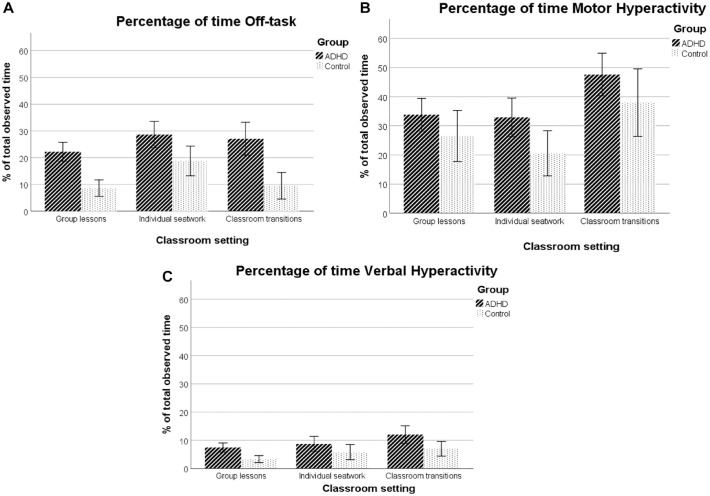
Bar graphs of the effects of classroom setting on Off-task (panel A), Motor Hyperactivity (panel B) and Verbal Hyperactivity (panel C) for the three settings observed. *Note*. Error bars reflect 95% confidence intervals. The Classroom Setting scale did not include mutually exclusive variables (the Other category is not depicted here).

Table 3 displays results of the multilevel analyses examining effects of group, setting and group by setting for percentage of time children were off-task and showed motor or verbal hyperactivity. Figure 1 visualizes for both groups the proportion of time each of these behaviors were shown during group lessons, individual seatwork and classroom transitions. For all, but one, of the models, the levels school and classroom did not improve the model fit. Hence, these models were reduced to two levels (observations clustered in students). Time spent in classroom transitions was much shorter than time spent in group lessons and individual seatwork (11.79% vs. 50.29% and 43.50%), with the ADHD and control group spending similar proportions of time in each setting (see Table 2).

**Table 3.** Group Differences on Behavioral Outcomes Across Settings as Measured With Classroom Observations.

	ADHD (*n* = 55)	Control (*n* = 34)	Effect of group	Effect of setting	Effect of group × setting
Off-task percentage			*B* = −10.51, *SE* = 4.81, *p* = .029	*B* = −2.20, *SE* = 1.20, *p* = .066	*B* = −1.58, *SE* = 1.97, *p* = .422
			ADHD > control		
Group	22.22 (12.78)	8.62 (8.69)			
Individual	28.64 (17.59)	18.79 (15.70)			
Transition	27.11 (22.16)	9.52 (13.13)			
Motor hyperactivity percentage			*B* = −6.85, *SE* = 7.41, *p* = .355	*B* = 7.02, *SE* = 1.19, *p* < .001	*B* = −1.70, *SE* = 3.10, *p* = .583
				TR>GR, IN	
Group	33.79 (20.29)	26.48 (24.84)			
Individual	32.90 (23.57)	20.56 (21.89)			
Transition	47.61 (26.33)	37.96 (30.70)			
Verbal hyperactivity percentage^a^			*B* = −3.03, *SE* = 2.46, *p* = .217	*B* = 2.29, *SE* = .64, *p* < .001	*B* = −.47, *SE* = 1.05, *p* = .653
				TR > GR, IN	
Group	7.45 (5.79)	3.35 (3.43)			
Individual	8.82 (9.59)	5.82 (7.57)			
Transition	12.02 (11.16)	7.00 (6.87)			

*Note*. Reported figures indicate *M*(SD).

GR = group lessons; IN = individual seatwork; TR = classroom transitions.

a Level classroom was included in the model.

When comparing the three classroom settings, a significant effect of setting was obtained for motor and verbal hyperactivity: Levels of motor and verbal hyperactivity were higher during classroom transitions than during individual seatwork and group lessons (for motor hyperactivity: *B* = −15.87, *SE* = 2.92, *p* < .001; *B* = −13.11, *SE* = 2.89, *p* < .001, respectively; for verbal hyperactivity; *B* = −2.61, *SE* = 1.03, *p* = .011; *B* = −4.29, *SE* = 1.02, *p* < .001, respectively). No differences were observed for motor and verbal hyperactivity during group lessons compared to individual seatwork (*B* = −2.76, *SE* = 2.87, *p* = .336; *B* = 1.68, *SE* = 1.01, *p* = .097, respectively). Although levels of motor and verbal hyperactivity seem higher in the ADHD and control group across settings (see Figure 1), group effects were not significant for neither of the two variables. Further, the non-significant group by setting interactions indicated that the effects of setting did not differ between the ADHD and control group.

For the proportion of time children were off-task, an effect of group was obtained, indicating that children in the ADHD group were more off-task than controls across settings. The effect of setting just escaped conventional levels of statistical significance for proportion of time children were off-task (*p* = .066). Exploratory post-hoc comparisons between settings revealed that children were more off-task during individual seatwork compared to group lessons (*B* = 8.13, *SE* = 1.80, *p* < .001), and transitions (*B* = 4.99, *SE* = 1.83, *p* = .006). Children were more off-task during classroom transitions compared to group lessons, although this effect also escaped conventional levels of significance (*B* = 3.14, *SE* = 1.81, *p* = .082). The group by setting interaction for off-task behavior was not significant, indicating that children in the ADHD group and controls were not differentially affected by setting.

Sensitivity analyses were performed in the groups matched on sex (*n* = 27 per group, with 17 boys and 10 girls per group), see Supporting Information Table S1. Results were similar to effects obtained in the full sample with significant and trend significant effects being replicated, with the exception of the trend significant effect of setting for off-task behavior that was no longer trend significant (*p* = .132).

### Additional Analyses Concerning Diagnostic Status

We explored whether the results were similar when comparing those children in the ADHD group that did not receive a clinical diagnosis of ADHD (according to parental report) (*n* = 41) to the control group (*n* = 34), which was the case, see Supplemental Table S2. In addition to the results reported with the full sample, there was also a significant effect of setting observed for off-task behavior, indicating that all children were less off-task during group lessons compared to individual seatwork and classroom transitions (*B* = 9.49, *SE* = 2.58, *p* < .001; *B* = 6.38, *SE* = 2.52, *p* = .011, respectively). Again, no group by setting interactions were observed.

## Discussion

This is the first study that systematically investigated the role of classroom setting, including classroom transitions, on ADHD behavior of children with ADHD and typically developing children using classroom observational measures. Children in the ADHD group were more off-task than typically developing children across settings. Inattention as well as (motor and verbal) hyperactivity of both groups were similarly affected by setting. Results showed that motor as well as verbal hyperactivity of all children increased during classroom transitions as compared to group lessons or individual seatwork.

Higher levels of motor movements and talking during classroom transitions may, for example, be explained by children walking around to get books for their next subject (reflected by an increase in motor hyperactivity) or by children asking questions about the instructions provided (reflected by an increase in verbal hyperactivity). However, the possible delays during transitions, together with the high demands placed on self-regulatory abilities (e.g., multiple instructions, switching between tasks) and motivation, may also have driven the increases in hyperactivity (Sonuga-Barke et al., 2008; Sonuga-Barke, Bitsakou, & Thompson, 2010). Nevertheless, we saw no evidence that transitions resulted in more profound hyperactivity in the ADHD group compared to controls. Our results differ from the study by Imeraj et al. (2016), that showed that the increase in hyperactivity during idle time (i.e., moments in which children were waiting) as compared to non-idle time was larger in the ADHD group than in the control group. These inconsistent findings may be explained by the type of setting studied by Imeraj et al. (2016) and in the current study, that is, idle time and classroom transitions, respectively. During idle time there is likely less stimulation compared to classroom transitions (e.g., where instructions are given) which may have a more profound detrimental effect on hyperactivity in children with ADHD as compared to the control group (Antrop et al., 2005). Indeed, in previous studies, lower levels of stimulation have been found to result in increased motor as well as verbal hyperactivity (Zentall & Zentall, 1983). In the current study idle time was not included in the analyses as the length of this setting was too short (on average <3.5% of total observation duration).

Based on research in typically developing children, it has been suggested that limiting time spent on transitions between activities could reduce hyperactivity and increase student engagement as well as time spent on learning activities (Arlin, 1979; Cameron et al., 2005; Codding & Smyth, 2008). In order to improve efficiency of classroom transitions, teachers may use behavioral strategies as effective instructions, monitoring of child behavior and feedback on (un)desired behavior (McIntosh et al., 2004). For children with ADHD, however, such transitions may also provide opportunities for short moments of movements as they experience difficulties staying seated or being silent (APA, 2013). There is evidence that motor movements have a functional role to counteract inattention in children with ADHD (Rapport et al., 2009; Sarver et al., 2015). In this cross-sectional study we did not observe a decrease in inattentive behavior during transitions as compared to other settings, but potential causal relationships have not yet been studied in classroom settings. Further research is needed to conclude on whether increased motor and verbal hyperactivity during transitions should be interpreted as problematic, and whether reducing transition times has beneficial effects for children with ADHD.

No setting by group interactions were observed, indicating that children with ADHD and typically developing children are similarly affected by classroom setting. This may be related to the fact that we did not collect data, and therefore lack insight, on whether groups differ on relevant parameters regarding child (e.g., self-regulation and motivational skills), teacher (e.g., levels of teacher supervision, number of delays) and environmental (e.g., availability of role models) factors that may affect classroom functioning. Although children with ADHD show impairments in neurocognitive domains related to self-regulation and motivation when compared to typically developing children, there is large heterogeneity in the type and severity of impairments within the ADHD group (Faraone et al., 2015). It may be that children with specific neurocognitive impairments are more vulnerable to specific classroom settings. For example, children that experience difficulties motivating themselves may struggle more with individual seatwork than group lessons. Another possibility that may explain the non-significant group by setting effects may be that teachers provide more supervision to children with ADHD to regulate (or compensate for) their self-regulation and motivational impairments, resulting in smaller differences between groups, especially during individual seatwork. However, the study by Imeraj et al. (2013) showed that children with ADHD remained more off-task than typically developing peers also after controlling for teacher supervision. Furthermore, smaller classes provide more possibilities for teachers to direct children’s behavior and is thus expected to be beneficial for children (Finn et al., 2003). This seems in particular important for children that experience difficulties regulating and motivating themselves, such as children with ADHD (Baker et al., 2008; Hart et al., 2011). By taking child, teacher and environmental factors into account, future studies may provide insight into whether these factors impact the relation between classroom setting and ADHD behaviors of children.

There was some evidence for a relation between setting and off-task behavior (regardless of ADHD), although this effect of setting escaped conventional levels of significance. Children seem off-task for a larger proportion of time during individual seatwork compared to group lessons, which is in contrast to predominantly older studies showing effects in the opposite direction (Lauth et al., 2006; Rimm-Kaufman et al., 2005; Steiner et al., 2014; Zentall, 1980). These disparate findings may be explained by the fact that in the past years several advantages have been made in education to promote interactivity in group lessons, such as interactive white boards that have a positive effect on students’ attention (see for review; Digregorio & Sobel-Lojeski, 2010). It might be possible that group lessons of teachers in this study included interactive lessons, resulting in less off-task behavior for all children (Baker et al., 2008). However, this finding may also relate to (one of) the abovementioned child, teacher, and/or environmental factors, such as the demand placed on self-regulation and intrinsic motivation by teachers during individual seatwork compared to group lessons, and the child’s ability to deal with this. Further research on the potential beneficial effects of group lessons on on-task behavior is thus needed.

Our current results should be interpreted in light of some limitations. One limitation may be that teachers use different techniques to regulate the child’s environment in lower and higher grades (Owens et al., 2018), and we would have needed a larger sample to study effects of grade. For example, in higher grades children are required to work more autonomous, while this may be challenging for children with ADHD because of their neurocognitive impairments. This may lead to an exacerbation of inattentive and/or hyperactive behavior in this group as compared to typically developing children, which could not have been observed in the current study because we did not differentiate between grades. Next, although the GUCCI is a reliable coding inventory to compare children with and without ADHD (Staff, Oosterlaan, Imeraj, et al., 2020), its convergent and divergent validity is limited. This is, however, found for many observational instruments (Staff, Oosterlaan, van der Oord, et al., 2020) and may be explained by the fact that impulsivity is not included in the GUCCI and/or that inattention scales of rating scale or interview measures are not restricted to off-task behavior (e.g., items on making careless mistakes, losing stuff). Nevertheless, low validity may have jeopardized the integrity of the current findings.

Taken together, this study showed that children with ADHD were similarly affected by classroom settings compared to their typically developing peers, despite being more off-task across settings. All children, regardless of ADHD, showed more motor and verbally hyperactive behaviors during classroom transitions compared to group lessons and individual seatwork. Further research is needed to answer whether this should be considered beneficial or problematic for children, particularly for children with ADHD.

## Supplemental Material

sj-docx-1-jad-10.1177_10870547231167522 – Supplemental material for The Relation Between Classroom Setting and ADHD Behavior in Children With ADHD Compared to Typically Developing Peers Click here for additional data file. Supplemental material, sj-docx-1-jad-10.1177_10870547231167522 for The Relation Between Classroom Setting and ADHD Behavior in Children With ADHD Compared to Typically Developing Peers by Anouck I. Staff, Jaap Oosterlaan, Saskia van der Oord, Barbara J. van den Hoofdakker and Marjolein Luman in Journal of Attention Disorders

sj-docx-2-jad-10.1177_10870547231167522 – Supplemental material for The Relation Between Classroom Setting and ADHD Behavior in Children With ADHD Compared to Typically Developing Peers Click here for additional data file. Supplemental material, sj-docx-2-jad-10.1177_10870547231167522 for The Relation Between Classroom Setting and ADHD Behavior in Children With ADHD Compared to Typically Developing Peers by Anouck I. Staff, Jaap Oosterlaan, Saskia van der Oord, Barbara J. van den Hoofdakker and Marjolein Luman in Journal of Attention Disorders
