# Supplementary material for: The Relation Between Classroom Setting and ADHD Behavior in Children With ADHD Compared to Typically Developing Peers
Source: J Atten Disord. 2023 Apr 11;27(9):939–50. doi: 10.1177/10870547231167522 (PMC10291114; doi:10.1177/10870547231167522)
Supplement: sj-docx-1-jad-10.1177_10870547231167522 – Supplemental material for The Relation Between Classroom Setting and ADHD Behavior in Children With ADHD Compared to Typically Developing Peers [file sj-docx-1-jad-10.1177_10870547231167522.docx]

**Supporting information.**

| **Table S1.** Group differences on behavioral outcomes across settings as measured with classroom observations in a subsample of groups matched on sex and age. | | | | | |
| --- | --- | --- | --- | --- | --- |
|  | ADHD  (*n*=27) | Control  (*n*=27) | Effect of group | Effect of setting | Effect of group x setting |
| *Off-task percentage* | |  | ***B*=-12.47, *SE*=6.20, *p*=.044** | *B*=2.67, *SE*=1.77, *p*=.132 | *B*=-1.11, *SE*=2.59, *p*=.669 |
|  |  |  | ADHD>control |  |  |
| Group | 24.54(10.60) | 8.35(8.51) |  |  |  |
| Individual | 29.54(20.50) | 20.77(16.61) |  |  |  |
| Transition | 30.32(23.13) | 10.99(14.47) |  |  |  |
| *Motor hyperactivity percentage* | | | *B*=-9.43, *SE*=8.81, *p*=.285 | ***B*=8.67, *SE*=2.61, *p*=.001** | *B*=-1.27, *SE*=3.81, *p*=.740 |
|  |  |  |  | TR>GR, IN |  |
| Group | 32.34(17.22) | 21.66(18.63) |  |  |  |
| Individual | 30.70(22.81) | 18.94(19.90) |  |  |  |
| Transition | 49.80(24.47) | 37.14(31.17) |  |  |  |
| *Verbal hyperactivity percentage ^a^* | | | *B*=-.08, *SE*=3.06, *p*=.980 | ***B*=3.15, *SE*=.94, *p*=.001** | *B*=-1.37, *SE*=1.37, *p*=.316 |
|  |  |  |  | TR>GR, IN |  |
| Group | 6.63(5.53) | 3.29(3.63) |  |  |  |
| Individual | 9.35(10.72) | 6.37(8.24) |  |  |  |
| Transition | 12.99(13.21) | 6.83(6.22) |  |  |  |
| *Note*. Reported figures indicate *M*(SD). GR=group lessons; IN=individual seatwork; TR=classroom transitions.  *^a^* Level classroom was included in the model. | | | | | |
