# Supplementary material for: The Relation Between Classroom Setting and ADHD Behavior in Children With ADHD Compared to Typically Developing Peers
Source: J Atten Disord. 2023 Apr 11;27(9):939–50. doi: 10.1177/10870547231167522 (PMC10291114; doi:10.1177/10870547231167522)
Supplement: sj-docx-2-jad-10.1177_10870547231167522 – Supplemental material for The Relation Between Classroom Setting and ADHD Behavior in Children With ADHD Compared to Typically Developing Peers [file sj-docx-2-jad-10.1177_10870547231167522.docx]

**Supporting information.**

| **Table S2.** Group differences on behavioral outcomes across settings as measured with classroom observations in a subsample of children without a clinical ADHD diagnosis compared to controls. | | | | | |
| --- | --- | --- | --- | --- | --- |
|  | ADHD  (*n*=41) | Control  (*n*=34) | Effect of group | Effect of setting | Effect of group x setting |
| *Off-task percentage* | |  | ***B*=-10.18, *SE*=5.06, *p*=.044** | ***B*=3.22, *SE*=1.37, *p*=.019** | *B*=-2.60, *SE*=2.07, *p*=.209 |
|  |  |  | ADHD>control | TR, IN>GR |  |
| Group | 22.35(13.31) | 8.62(8.69) |  |  |  |
| Individual | 31.40(18.16) | 18.79(15.70) |  |  |  |
| Transition | 29.42(21.88) | 9.52(13.13) |  |  |  |
| *Motor hyperactivity percentage* | | | *B*=-8.06, *SE*=7.84, *p*=.304 | ***B*=5.64, *SE*=2.11, *p*=.008** | *B*=-.33, *SE*=3.18, *p*=.916 |
|  |  |  |  | TR>GR, IN |  |
| Group | 32.26(22.74) | 26.48(24.84) |  |  |  |
| Individual | 34.05(25.39) | 20.56(21.89) |  |  |  |
| Transition | 43.23(25.11) | 37.96(30.70) |  |  |  |
| *Verbal hyperactivity percentage ^a^* | | | *B*=-2.13, *SE*=2.54, *p*=.402 | ***B*=2.84, *SE*=.72, *p*<.001** | *B*=-1.03, *SE*=1.09, *p*=.346 |
|  |  |  |  | TR>GR, IN |  |
| Group | 7.25(5.86) | 3.35(3.43) |  |  |  |
| Individual | 8.63(9.04) | 5.82(7.57) |  |  |  |
| Transition | 12.93(11.61) | 7.00(6.87) |  |  |  |
| *Note*. Reported figures indicate *M*(SD). GR=group lessons; IN=individual seatwork; TR=classroom transitions.  *^a^* Level classroom was included in the model. | | | | | |
